# Frequency of Circulating CD4^+^Ki67^+^HLA-DR^−^ T Regulatory Cells Prior to Treatment for Multidrug Resistant Tuberculosis Can Differentiate the Severity of Disease and Predict Time to Culture Conversion

**DOI:** 10.3389/fimmu.2018.02438

**Published:** 2018-10-25

**Authors:** Selena Ferrian, Melinda Ross, Francesca Conradie, Shaheed Vally Omar, Nazir Ismail, Francesca Little, Gilla Kaplan, Dorothy Fallows, Clive M. Gray

**Affiliations:** ^1^Division of Immunology, Department of Pathology, Institute of Infectious Disease and Molecular Medicine, University of Cape Town, Cape Town, South Africa; ^2^Department of Statistical Sciences, University of Cape Town, Cape Town, South Africa; ^3^Faculty of Health Sciences, University of the Witwatersrand, Johannesburg, South Africa; ^4^Centre for Tuberculosis, National Institute of Communicable Diseases, Johannesburg, South Africa; ^5^Department of Medical Microbiology, University of Pretoria, Pretoria, South Africa; ^6^Public Health Research Institute, Rutgers University, Newark, NJ, United States; ^7^National Health Laboratory Services, Cape Town, South Africa

**Keywords:** Treg cells, *Mycobacteria tuberculosis*, multi-drug resistant tuberculosis, lung cavities, sputum culture, smear grades, culture conversion

## Abstract

Identifying a blood circulating cellular biomarker that can be used to assess severity of disease and predict the time to culture conversion (TCC) in patients with multidrug resistant tuberculosis (MDR-TB) would facilitate monitoring response to treatment and may be of value in the design of future drug trials. We report on the frequency of blood Ki67^+^HLA-DR^−^ CD4+ T regulatory (Treg) cells in predicting microbiological outcome before initiating second-line treatment for MDR-TB. Fifty-one patients with MDR-TB were enrolled and followed over 18 months; a subset of patients was sputum culture (SC) negative at baseline (*n* = 9). SC positive patients were divided into two groups, based on median TCC: rapid responders (≤71 days TCC; *n* = 21) and slow responders (>71 days TCC; *n* = 21). Whole blood at baseline, months 2 and 6 was stimulated with M tuberculosis (Mtb) antigens and Treg cells were then identified as CD3^+^CD4^+^CD25^hi^FoxP3^+^CD127^−^CD69^−^ and further delineated as Ki67^+^HLA-DR^−^ Treg. The frequency of these cells was significantly enlarged at baseline in SC positive relative to SC negative and smear positive relative to smear negative patients and in those with lung cavitation. This difference was further supported by unsupervised hierarchical clustering showing a significant grouping at baseline of total and early differentiated memory Treg cells in slow responders. Conversely, there was a clustering of a lower proportion of Treg cells and activated IFNγ-expressing T cells at baseline in the rapid responders. Examining changes over time revealed a more gradual reduction of Treg cells in slow responders relative to rapid responders to treatment. Receiver operating curve analysis showed that baseline Mtb-stimulated Ki67^+^HLA-DR^−^ Treg cells could predict the TCC of MDR-TB treatment response with 81.2% sensitivity and 85% specificity (AUC of 0.87, *p* < 0.0001), but this was not the case after 2 months of treatment. In conclusion, our data show that the frequency of a highly defined Mtb-stimulated blood Treg cell population at baseline can discriminate MDR-TB disease severity and predict time to culture clearance.

## Introduction

Multidrug resistant tuberculosis (MDR-TB) is a major global concern and none more than in Africa, where the HIV epidemic in numerous countries fuels enhanced TB disease, including the increasing burden of MDR and XDR-TB, and accelerated mortality in these patients (1, 2). TB disease induces a state of immune activation in the infected host, and increased expression of activation markers on circulating T cells in blood from patients with active TB has been described (3). Circulating regulatory CD4^+^CD25^+^FoxP3^+^ T cells (Treg) are involved in the regulation of self-tolerance, autoimmunity, and suppression of immune responses during infections (4). These cells are increased in both drug-sensitive- and MDR-TB, where they are associated with high bacillary burden (5–7) and are possibly induced by chronic inflammation (8). Treg cells not only accumulate in the blood circulation, but also at sites of disease, for example in pleural, ascetic, pericardial, or bronchoalveolar fluids (7, 9), where high levels of SOCS and IRAK-M (intracellular) and IL-10 and TGF-βRII, IL-1Rn, and IDO (extracellular) immune suppressive mediators (10) would paralyze Th1-mediated protective immunity (11,–16). The suppression of potentially protective anti-TB immunity and the short-range action of these soluble suppressive mediators could result in unchecked bacterial replication and renders the host to unregulated inflammation and the spread of pathology. This is a seemingly counter-intuitive involvement of Treg cells in TB pathology. Different subsets of Treg cells have been identified in patients with drug susceptible TB (16), where mycobacterial antigen-induced expansion of Ki67-expressing CD4^+^FoxP3^+^ Treg cells have been shown in patients with active pulmonary mTB that persist into MDR-TB disease (17). It is possible that the prolonged exposure of mycobacterial antigens in patients with MDR-TB may result in the continued proliferation of Treg cells. We previously described several inflammatory plasma markers in MDR-TB patients (18) that could predict microbiological outcome and showed that chronic inflammation at baseline existed in MDR-TB patients who responded slowly to second-line antibiotics. In the present study, we focused on Treg cells and hypothesized that the chronic inflammation observed in our MDR-TB patients (18), along with lung cavitation, was associated with elevated proportions of these cells and are useful biomarkers of disease severity and treatment response rates.

We defined Treg cells according to the consensus “essential marker set” for suppressive Tregs: CD3^+^CD4^+^CD25^hi^FoxP3^+^CD127^−^ (19). As both FoxP3 and CD25, classic markers of Treg cells, are upregulated on activated non-suppressive T cell subsets (20), it was critical to define the population as being CD25 bright and CD127 negative. We further used more stringent criteria to exclude acutely activated T cells and cells recently emigrated from lymphoid tissues (21), such as the lung (22), by defining cells as CD69 negative. Chronically activated HLA-DR expressing T cells were also factored out by defining the Treg cells after Mtb stimulation as HLA-DR negative. We wished to include actively proliferating cells by focusing on Ki67-expression (23) and our defined Treg subset, CD3^+^CD4^+^CD25^hi^FoxP3^+^CD127^−^CD69^−^Ki67^+^HLA-DR^−^, was able to discriminate between sputum culture (SC) negative and positive and those with and without lung cavitation.

Additionally, the presence of these proliferating Tregs, in response to whole blood Mtb antigen re-stimulation, could also predict the time to culture conversion (TCC) and thus serve as a marker of microbiological outcome.

## Materials and methods

### Cohort description

A total of 51 HIV negative patients (Table 1) initiating treatment for MDR-TB at Sizwe Hospital in Johannesburg, South Africa were enrolled in the study, which has previously been described (18). Diagnosis of MDR-TB was based on SC (Bactec MGIT 960, Beckton Dickinson, Baltimore, MD) and phenotypic drug susceptibility testing (DST; Bactec MGIT 960) and/or genotypic resistance by PCR (Line Probe Assay, Hain Lifesciences). Baseline (enrollment) clinical and microbiological characteristics are summarized for many of the enrolled patients (18). Nine study participants were SC negative at the time of enrollment; all of these patients were SC positive at a median of 55 days (IQR: 16.5–87.5) prior to baseline, suggesting that the infecting TB was partially responsive to first-line drugs. Eight of the culture negative patients (88.9%) had received prior TB treatment, and all were sputum smear negative. A wide diversity in body mass index (BMI) was noted, with an overall median of 17.0, IQR: 17.0–22.5, with 20 patients (39%) classified as underweight (<18.5), 20 (39%) as normal BMI (18.5–24.9), 5 (8%) as overweight (25.0–29.9) and one patient classified as obese (>30), based on WHO criteria.

**Table 1:** Baseline smear data, lung cavitation, body mass index, age, and gender in study participants showing sputum culture status and response to MDR-TB treatment.

**Covariate**	**%Sputum culture negative (*n* = 9)**	**%Rapid Responder (*n* = 21)**	**%Slow Responder (n = 21)**	***P*-value**
Baseline smear grades (%)				0.0008
0	100	36	6.0	
1	0	5.1	6.0	
2	0	23.7	16.0	
3	0	5.1	6.0	
4		30.5	66.0	
Baseline cavitation (%)				0.08
Cavitation	78	62.5	86.4	
No cavitation	22	37.5	13.6	
Body mass index [median (IQR)]	21.1 (18–24)	19.2 (17–21)	17.6 (17–19)	0.12
Age [median (IQR)]	55 (40–58)	41 (27–50)	38 (25–49)	0.39
Gender (%)				0.13
Female	22	44.1	30.0	
Male	78	55.9	70.0	

### Drug susceptibility testing and bacteriological response to treatment

Sputum culture and AFB smear microscopy were performed at the time of admission (baseline, Table 1) and monthly thereafter, as part of routine monitoring. Sputum culture conversion was defined as two consecutive TB culture negative results, separated by at least 30 days, with no subsequent culture positive results. Time to culture conversion (TCC) was defined as the interval between the date of second-line treatment initiation and the date of collection of the sputum specimen that yielded the first negative culture result. There were 42 patients who were SC positive at enrollment and based on the median TCC in these patients, we categorized patients in this study as either slow responders (TCC ≥ 71 days) or fast responders (TCC < 71 days) for all subsequent analysis. DST (Bactec MGIT 960) was performed for all culture positive MDR-TB patients within 30 days of starting treatment.

### Assessment of disease severity

Disease severity was evaluated based on chest X-rays, performed at the time of admission to hospital and scored for extent of lung involvement and cavitation. Chest X-ray reviews were obtained from patient records and graded 0–4 (Table 1).

### Ethics

This study was carried out in accordance with the recommendations and approval of the Research Ethics Committees of the University of the Witwatersrand and University of Cape Town and the Institutional Review Board of Rutgers University (Pro0120090189). All study participants gave written informed consent in accordance with the Declaration of Helsinki.

### Flow cytometry analyses

QuantiFERON-TB Gold tubes consisting of a negative control (Nil antigen); Mtb tube [containing of a cocktail of ESAT-6, CFP-10, and TB7.7(p4) peptides]; Mitogen (Mito) tube, [containing phytohaemagglutinin (PHA) as the positive control] were obtained from Cellestis Inc. (Valencia, CA, USA) and used in the whole blood intracellular cytokine (ICS) assay. Tuberculin purified protein derivative (PPD) was obtained from the Staten Serum Institute (Copenhagen, Denmark) and used as a separate stimulus at 10 μg/ml in the whole blood ICS assay. The following fluorescently conjugated monoclonal antibodies were used in this study: anti-CD8 V500 (RPA-T8), anti–IFNγ Alexa Fluor 700 (B27), anti-TNFα PECy7 (MAb11), anti-Ki-67 FITC (B56) and anti-IL2 Brilliant Violet 605 (5344.111), anti-CD25 Brilliant Violet 421 (M-A251), anti-CD45RA PerCP-Cy5.5 (HI100), anti-CD69 PE (L78), anti-CD127 PE-CF594 (HIL-7R-M21), anti-FoxP3 Alexa Fluor 647 (259D/C7), Transcription Factor Buffer Set and Brilliant Stain buffer all from BD Biosciences (San Diego, CA, USA); anti-CD3 Brilliant Violet 650 (OKT3) and anti-HLA-DR APC-Cy7 (L243) from Biolegend (San Diego, CA, USA) anti-CD4 PECy5.5 (S3.5) from Invitrogen (Grand Island, NY, USA); anti-CD27 PECy5 (O323) from eBioscience (Wembley, UK); as summarized in Supplementary Figure 2A. Fluorescence Minus One (FMO) were performed to validate the expression of Ki67 in both rapid and slow responders (Supplementary Figure 2B). A total of approximately 2.5 million events were run through BD™ LRS II flow cytometer. Multiple-parameter flow cytometry data were analyzed using FlowJo software (v9.9; Tree star Inc., Ashland, OR, USA). The gating strategies for both regulatory T cells and T cell activation are presented in Supplementary Figures 1A,B, respectively. We further showed how gating on regulatory T cells positive for CD69 yields Th1 cytokines (Supplementary Figure 1C). Combinations of cytokine-producing cells were determined using Boolean gating in FlowJo, followed by further analysis using Pestle v1.6.2 and Spice v5.3 (Dr. Mario Roederer, Vaccine Research Center, National Institute of Allergy and Infectious Diseases, National Institutes of Health, Bethesda, MD, USA).

### Whole blood ICS assay

Within a maximum of 45 min after blood collection, 1 ml of sodium heparinized blood was incubated with PPD (10 μg/ml) or added to the QuantiFERON-TB Gold tubes in the presence of anti-CD28 (1 μg/ml, BD Pharmingen) and anti-CD49d (1 μg/ml, BD Pharmingen) for 7 h at 37°C. Blood incubated in the QuantiFERON-Nil Gold tube served as a negative control, and blood incubated in the QuantiFERON-Mitogen Gold tube as a positive control. After 7 h, brefeldin A (10 μg/ml; Sigma-Aldrich, Dorset, UK) was added, and the incubation continued for an additional 5 h (23). After 12 h incubation, 2 mM EDTA was added for 10 min, then RBC were lysed and white blood cells were fixed with FACS Lysing Solution (BD Biosciences), followed by cryopreservation in freezing medium containing 10% DMSO/90% fetal bovine serum and stored in liquid nitrogen until use. Cryopreserved cells were thawed, washed in PBS, permeabilized with Perm/Wash solution (BD Biosciences). Cells were stained with a panel of conjugated mAbs, described above, for 45 min at 4°C, washed in Perm/Wash Buffer (BD Biosciences), and resuspended in PBS containing 1% formaldehyde, prior to acquisition on an LSRII flow cytometer (BD Biosciences).

### Luminex multiplex immunoassay

Blood samples were collected on the day after patients started MDR-TB therapy and thereafter at 2, 4, and 6 months of treatment as previously described (18).

### Statistics

Ki67^+^HLA-DR^−^ Treg cells frequencies were log transformed prior to analysis for **Figures 2B, 3A, 3B, 4B, 4C, 4D**, Supplementary Figures 9 and 11. Missing values at follow up visits were replaced by mean values within each group. Missing values were either due to <50 Ki67^+^HLA-DR^−^ total Treg cells in the analysis gate or missed patient visits. Marker frequencies at different time points and for different groups were summarized using medians and interquartile ranges and illustrated using box-and-whisker plots. Differences between groups (SC, SS, and cavitation) were tested using the Mann-Whitney U-test. Where tests of differences involved more than two groups, a Kruskal-Wallis test was employed. Differences between fast and slow responders at each time point were tested using a Mann-Whitney U-test and between time points were tested using the Wilcoxon matched-pair sign rank test. Associations between two categorical variables were assessed using Fisher's Exact test and between two continuous variables using Spearman's Rank Correlation. Mixed effect linear regression models were fitted using maximum likelihood estimation on the unimputed data, thereby allowing for model-based imputation of the missing data. The models estimate and compare the changes from baseline to the months 2 and 6 visits for the two responder groups, while taking into account the within subject correlation of the repeated measurements. The models included covariates identified as possible confounders. Receiver Operating Characteristic (ROC) analysis was used to assess the predictive nature of Ki67^+^HLA-DR^−^ Treg cells with TCC. Observed *p*-values are reported throughout the paper for all tests carried out and no specific references are made to the use of a threshold value to determine statistical significance. Lower *p*-values are indicative of stronger associations and larger ones of weaker or no meaningful association. Analysis was carried out using R (24). A heat map was generated and clustering of differences were analyzed using Qlucore Omics Explorer 3.1 with interface to R (Olucore AB, Lund, Sweden).

## Results

### Baseline Mtb and PPD-stimulated Ki67^+^HLA-DR^−^ treg cells associate with disease severity and TCC

**Figure 1:**
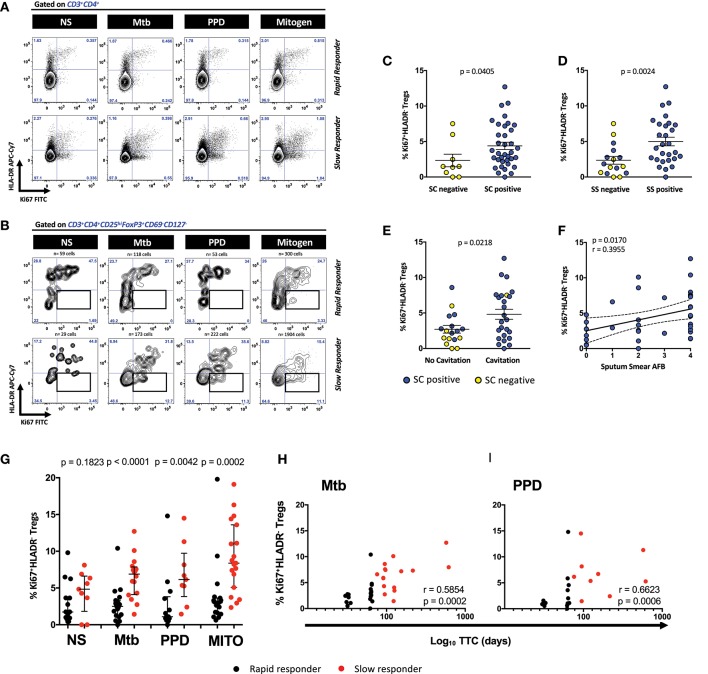
Defining CD4^+^ Ki67^+^HLA-DR^−^ Treg cells and the relationship with cavitation and microbiological outcomes. **(A)** Representative contour plots of Ki67/HLA-DR expression on CD3^+^CD4^+^ T cells from a rapid and slow responder to TCC. **(B)** Representative contour plots of Ki67/HLA-DR expression on CD4^+^CD25^hi^FoxP3^+^CD127^−^CD69^−^ Treg cells from a rapid and slow responder to TCC. The lower right quadrant (boxed area) shows the frequency of Ki67^+^HLA-DR^−^ Treg cells used in subsequent analysis. **(C)** Comparison of Ki67^+^HLA-DR^−^ Treg cells between sputum culture (SC) negative (*n* = 9) and SC positive patients (*n* = 36). Six SC+ patients were excluded from the analysis due to the numbers of Ki67^+^HLA-DR^−^ Treg cells being <50 in the analysis gate. The horizontal line represents the median values ± interquartile ranges (IQR). Yellow symbols represent SC negative patients and blue symbols represent SC positive patients. The Mann–Whitney *U*-test was used for statistical comparison. **(D)** Comparison of Ki67^+^HLA-DR^−^ Treg cells between sputum smear (SS) negative (*n* = 16) and SS positive patients (*n* = 28). Seven SC+ patients were excluded from the analysis due to the numbers of Ki67^+^HLA-DR^−^ Treg cells being <50 in the analysis gate. The horizontal line represents the median values ± interquartile ranges (IQR). Yellow symbols represent SC negative patients and blue symbols represent SC positive patients. The Mann–Whitney *U*-test was used for statistical comparison. **(E)** Comparison of Ki67^+^HLA-DR^−^ Treg cells between patients with no lung cavitation (*n* = 17) and patients with lung cavitation (*n* = 25). Nine SC+ patients were excluded from the analysis due to either the numbers of Ki67^+^HLA-DR^−^ Treg cells being <50 in the analysis gate (*n* = 6) and 3 with no X-ray data (not analyzed, NA). The horizontal line represents the median values ± interquartile ranges (IQR). Yellow symbols represent SC negative patients and blue symbols represent SC positive patients. Statistical comparisons were made using the Mann–Whitney *U*-test. **(F)** Fisher's exact test in SC+ patients between Ki67+HLA-DR^−^ Treg cells and sputum smear Acid Fast Bacilli (AFB) grades in Mtb-stimulated cells. Blue symbols represent SC positive patients. **(G)** Comparison of Ki67^+^HLA-DR^−^ Treg cells after short-term stimulation with Mtb antigens (*n* = 36), PPD (*n* = 23), mitogen (*n* = 38), and unstimulated NS (*n* = 25) cells between rapid responders (black symbol) vs. slow responders (red symbol). Six Mtb stimulated patients were excluded due to either <50 Treg cells in the analysis gate (*n* = 3), or missing samples (*n* = 3). Likewise, 19 patients were excluded due to either <50 Treg cells in the analysis gate (*n* = 9) or that there was no PPD tube available (*n* = 10). Four mitogen stimulated patients were excluded due to either <50 Treg cells in the analysis gate (*n* = 2), or missing samples (*n* = 2). Seventeen unstimulated patients were excluded due to either <50 Treg cells in the analysis gate (*n* = 15), or missing samples (*n* = 2). The horizontal line represents the median values ± interquartile ranges (IQR). Statistical comparisons between responding patients were made using the Mann–Whitney *U*-test. **(H,I)** Pearson correlation between Ki67^+^HLA-DR^−^ Treg cells and Log_10_ time to culture conversion (TCC) after Mtb and PPD, respectively. Black symbols represent rapid responders and red symbols represent slow responders.

Figures 1A,B show the expression of proliferating Ki67 positive and HLA-DR positive cells within the CD3^+^CD4^+^ T cells and the CD3^+^CD4^+^CD25^hi^FoxP3^+^CD127^−^CD69^−^ regulatory T cell population in two representative MDR-TB patients. Gates for regulatory T cell positive for Ki67 and HLA-DR were placed based on the total population of CD3^+^CD4^+^ T cells. Although the counts of Ki67^+^HLA-DR^+^ Treg cells in the tubes without exogenous stimuli (i.e., the no stimulus tube) were very low, those detected appeared to be spontaneously proliferating, suggesting that cells were entering mitosis from the *in vivo* state of the SC+ patients. However, it is clear that a more defined double expressing Ki67^+^HLA-DR^+^ population of Treg cells in the rapid responders exists, relative to the slow responders, where there is a more substantial population of proliferating Treg cells that are negative for HLA-DR (Figure 1B). By gating on these cells in all patients (*n* = 51), we identified that Mtb-stimulated Ki67^+^HLA-DR^−^ Treg cells were able to distinguish SC negative from positive (Figure 1C), sputum smear negative from positive (Figure 1D) and patients with and without lung cavitation (Figure 1E). The extent of disease severity in the SC positive patients (*n* = 42) was further assessed by the presence/absence of lung cavitation and smear grades. The correlation between cavitation and smear grades showed a positive trend line (*p* = 0.0980; *r* = 0.2761) and both variables were associated with patients responding slowly to treatment. Supplementary Figure 3 shows that a higher proportion of slow responders (16/18 patients, 88.8%) showed cavitation in the lung compared to rapid responders (12/20, 60%; *p* = 0.0673). Slow responders also showed higher baseline SS relative to rapid responders (*p* = 0.0071; *r* = 0.4142), where the majority of these patients at baseline displayed grade 4 SS (13/20, 65%,) and very few grade 0 SS (1/20, 5%). Conversely, rapid responders showed less grade 4 SS (6/21, 28.5%) and more grade 0 SS (7/21, 33.3%). This scenario suggests that bacillary load was likely driving cavitation in our patients. Further, when we compared the Ki67^+^HLA-DR^−^ Treg population frequency between patients who were SC positive with and without cavitation, the differences were not large (*p* = 0.29). No associations were found with either cavitation scores (*r* = 0.0493; *p* = 0.7852). However, when looking at the relationship between smear grade and Ki67^+^HLA-DR^−^ Tregs, a positive correlation was found in these patients (*r* = 0.3955; *p* = 0.0170, Figure 1F). We can thus conclude that the frequency of Ki67^+^HLA-DR^−^ Treg cells were more abundant in the blood of MDR patients with active TB disease.

When we examined for the presence of these cells in SC+ patients (*n* = 42) responding either rapidly (fast responders) or slowly (slow responders) to second line therapy, the frequency of Ki67^+^HLA-DR^−^ Treg cells significantly distinguished the pace of Mtb clearance (Figure 1G). Relative to the fast responder group, Mtb stimulation of whole blood gave rise to significantly more Ki67^+^HLA-DR^−^ Treg cells (*p* < 0.0001) in the slow responding group, mirrored along with unstimulated cells, PPD and mitogen stimulation (Figure 1G). When we regarded TTC as a continuous variable, we found significant correlations with the proportion of Mtb and PPD-stimulated Ki67^+^HLA-DR^−^ Treg cells (Figures 1H,I: *r* = 0.5854, *p* = 0.0002 and *r* = 0.6623, *p* = 0.0006, respectively). The significant binary Treg response to mitogen and unstimulated cells (used as positive and negative controls) weakened when correlated with TCC (*r* = 0.32, *p* = 0.052; *r* = 0.30, *p* = 0.215, respectively; Supplementary Figure 4), suggesting that the Treg differences found between fast and slow responders is likely to be specific to Mtb stimulation. When we compared Mtb and PPD-stimulated Ki67^+^HLA-DR^−^ Treg cells between SC-negative and SC-positive patients at baseline (Supplementary Figure 5), it was clear that Mtb-stimulated Ki67^+^HLA-DR^−^ Treg cells were significantly lower in patients who were SC negative. Thus, our data show that proliferating Treg cells are more abundant in the blood of patients with active TB disease where there is likely to be high amounts of antigenic stimuli from the lung, as witnessed by both the presence of smear and cultured bacteria.

To understand whether proliferative-competent Treg cells was a characteristic associated with microbiological outcome, we examined whether non-proliferating Mtb-stimulated Ki67^−^HLA-DR^−^ (double negative) regulatory T cells could discriminate between responder groups. Supplementary Figure 6 shows that this double negative subset of regulatory T cells was significantly higher in the SC-positive compared to the SC-negative group but could not distinguish between fast and slow responders (Supplementary Figure 7). We further verified whether our results were due to an increase of the Treg bulk from which this subset of Tregs derive. No correlations were found between Tregs and Ki67^+^HLA-DR^−^ Tregs in either TB (*p* = 0.4264; *r* = −0.1368) or PPD (*p* = 0.5364; *r* = −0.1359) stimulated cells. However, an inverse correlation was found between the Treg bulk and TCC (Supplementary Figure 9). As summarized in Supplementary Figure 10, slow responders had higher frequencies of Ki67^+^HLA-DR^−^ Tregs but lower frequencies of Tregs (Supplementary Figure 10). These data thus confirm the ability of Ki67^+^HLA-DR^−^ Treg cells to provide discrimination between the swiftness of bacterial clearance to second-line antibiotic treatment.

### Higher frequencies of baseline early differentiated (ED) memory Ki67^+^HLA-DR^−^ treg are found in slow vs. fast responders

**Figure 2:**
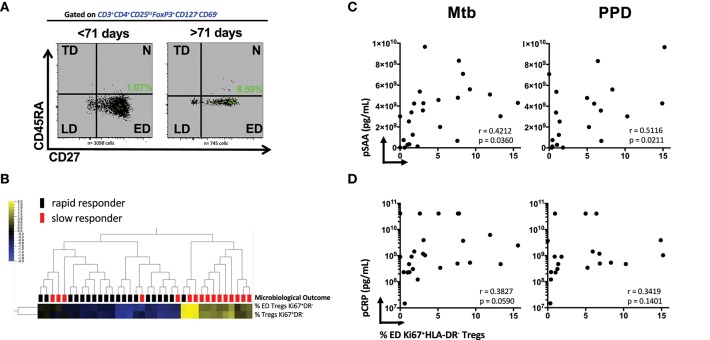
Early Differentiated (ED) memory CD4+ Ki67^+^HLA-DR^−^ Treg cells and the relationship between microbiological outcome and plasma inflammatory mediators. **(A)** Representative density plots showing the frequency of ED (CD27^+^CD45RA^−^) Mtb stimulated Ki67^+^HLA-DR^−^ Treg cells (green dots) overlayed on total memory populations. **(B)** Unsupervised hierarchical clustering of ED and total memory Treg cells with microbiological outcome. Solid black blocks are rapid responders (*n* = 20) and red blocks are slow responders (*n* = 16). Six patients were excluded from the analysis. Yellow represents up-regulated cell frequency and blue represents down-regulated. **(C,D)** Pearson correlations between ED (CD27^+^CD45RA^−^) Mtb- and PPD- stimulated Ki67^+^HLA-DR^−^ Treg cells and plasma concentration of SAA and CRP.

We next investigated whether the stage of memory maturation of Treg cells was associated with the rapidity of TCC. Figure 2A shows a representative overlay of Mtb-stimulated Ki67^+^HLA-DR^−^ memory Treg cells, where the ED memory phenotype was typically more abundant in slow responders (>71 days). This was collectively observed in Supplementary Figures 8A,B, where the frequency of Mtb and PPD stimulated ED memory Treg at baseline was significantly higher in those responding more slowly to treatment. This was further reflected by a positive correlation with TCC (Supplementary Figures 8C,D, *p* < 0.0001 and *p* = 0.0279, respectively) and confirmed (Figure 2B) with unsupervised hierarchical clustering (with a false discovery rate, *q* = 0.05) showing there was a significant grouping at baseline of total and ED memory Ki67^+^HLA-DR^−^ Treg cells in patients responding slowly to treatment. These data suggest that functionally active Ki67^+^HLA-DR^−^ Treg cells are in the early stages of memory maturation.

We recently showed that SAA and CRP were among predictive markers of TCC (18) from some of the same participants in the cohort. The significant positive association of ED Ki67^+^HLA-DR^−^ Treg cells with Mtb (*r* = 0.4212, *p* = 0.0360) and PPD (*r* = 0.5116, *p* = 0.0211, Figure 2C) in patients who were SC positive shows that SAA levels in sub-optimally treated MDR-TB patients is associated with the elevated Treg cell population at baseline. Although not as significant, plasma CRP levels trended in the same manner (Figure 2D). Taken together, these data suggest that the more inflammation in the host with active MDR-TB disease, the higher the frequency of Treg cells that recognize Mtb and the slower TCC. These patients were also more likely to be smear positive and thus higher levels of bacterial load and plasma acute phase proteins may provide a plausible connection between inflammation, bacillary load, disease severity (as measured by lung cavitation, Figures 1D,E and Supplementary Figure 3) and the higher frequency of Treg cells in these patients.

### Associations between treg and activated Mtb-stimulated T cell subsets and microbiological outcome

**Figure 3:**
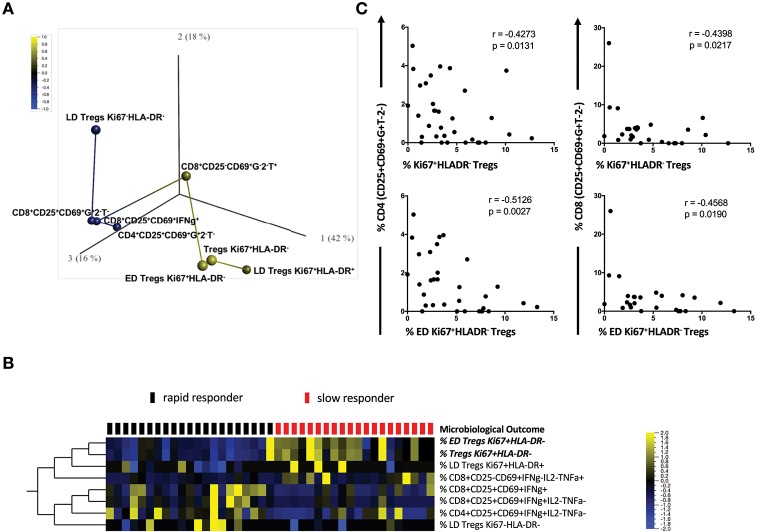
Correlation network of T cell subsets between slow and rapid responders. **(A)** Rank-regression analysis of PCA-transformed data showing a correlation network of the T cell phenotypes associated with TCC. T cell subsets positively correlating with slow responders are shown in yellow and those negatively correlating with a slow response to TCC are shown in blue. The distance between colored circles represent the strength of association. **(B)** Hierarchical clustering heat map of different CD4 and CD8 Treg phenotypes [with a FDR (q) of 0.05], supervised by microbiological outcome (TCC). Red blocks represent slow responders (*n* = 20) and black blocks represent rapid responders (*n* = 21) with yellow indicating increased frequency of Treg cells, blue a lowered frequency and black no difference between responder groups. (**C**) Pearson correlations between activated CD4^+^/CD8^+^ T cells and regulatory Ki67^+^HLA-DR^−^ Treg cells and ED CD27^+^CD45RA^−^Ki67^+^HLA-DR^−^ Treg cells Mtb- stimulated whole blood.

The complexity of our multicolor panel allowed us to identify how different Mtb-stimulated T cell subsets were associated with each other and with TCC. We conducted a rank-regression analysis applying this to PCA-transformed data to identify a correlation network. Figure 3A shows a PCA analysis of the T cell phenotypes that were associated with the timing of TCC. T cell subsets positively correlated with slow responders are shown in yellow (ED Tregs Ki67^+^HLA^−^DR^−^; LD Treg Ki67^+^HLA-DR^+^; Tregs Ki67^+^HLA-DR^−^; CD8^+^CD25^−^CD69^+^IFNγ^−^IL-2^−^TNFa^+^) and those negatively correlated with a slow response to TCC are show in blue (LD Tregs Ki67^−^HLA-DR^−^; activated IFNγ-producing CD4, and CD8 T cells: CD4^+^CD25^+^CD69^+^IFNγ^+^IL-2^−^TNFa^−^, CD8^+^CD25^+^CD69^+^IFNγ^+^IL-2^−^TNFa^−^; CD8^+^CD25^+^CD69^+^IFNγ^+^. In this analysis, both total Ki67^+^HLA-DR^−^ and ED Ki67^+^HLA-DR^−^ Treg memory cells were most strongly positively correlated with delayed TCC (*r* = 0.584, *p* = 6.1 × 10^−5^ and *r* = 0.548, *p* = 0.0002; respectively). LD Ki67^+^HLA-DR^+^ Treg cells and CD8^+^CD25^−^CD69^+^IFNγ^−^IL-2^−^TNFa^+^ were positively correlated with delayed TCC, but with lower r values (*r* = 0.312, *p* = 0.046 and *r* = 0.371, *p* = 0.017; respectively), which made them lessor candidates for predicting TCC. The inverse relationship between activated T cells: CD4^+^CD25^+^CD69^+^IFNγ^+^IL-2^−^TNFα^−^ (*r* = −0.322, *p* = 0.039); CD8^+^CD25^+^CD69^+^IFNγ^+^ (*r* = −0.371, *p* = 0.016); CD8^+^CD25^+^CD69^+^ IFNγ^+^IL-2^−^TNFα^−^ (*r* = −0.360, *p* = 0.020); and LD Treg Ki67^+^HLA-DR^+^ (*r* = −0.332, *p* = 0.033) and delayed TCC also makes these cell populations possible candidates as predictors of TCC. These associations were further reflected when we conducted a supervised hierarchical clustering of Treg cell populations, inputting missing values for the three patients with counts below 50 total Treg cells (Figure 3B). We showed that of all the possible population marker permutations, both ED Tregs Ki67^+^HLA-DR^−^ (*q* = 0.019) and Tregs Ki67^+^HLA-DR^−^ (*q* = 0.011) were upregulated at baseline in the majority of slow responders to treatment after accounting for false discovery rates. It was noteworthy that there was up-regulation of activated CD4^+^ and CD8^+^ T cells in rapid responders, with increased expression of CD25^+^CD69^+^IFNγ^+^ but negative for IL-2 and TNFα (Figure 3B). Additionally, Ki67^+^HLA-DR^−^ and ED Ki67^+^HLA-DR^−^ Tregs were inversely correlated with both activated CD4^+^CD25^+^CD69^+^IFNγ^+^IL-2^−^TNFa^−^ and CD8^+^CD25^+^CD69^+^IFNγ^+^IL-2^−^TNFa^−^ cells (Figure 3C), suggesting a possible suppressive role of ED Ki67^+^HLA^−^DR^−^ Tregs on mTb stimulated IFNg-expressing T cells.

### Changes in Mtb-stimulated Ki67^+^HLA-DR^−^ treg cells over time

**Figure 4:**
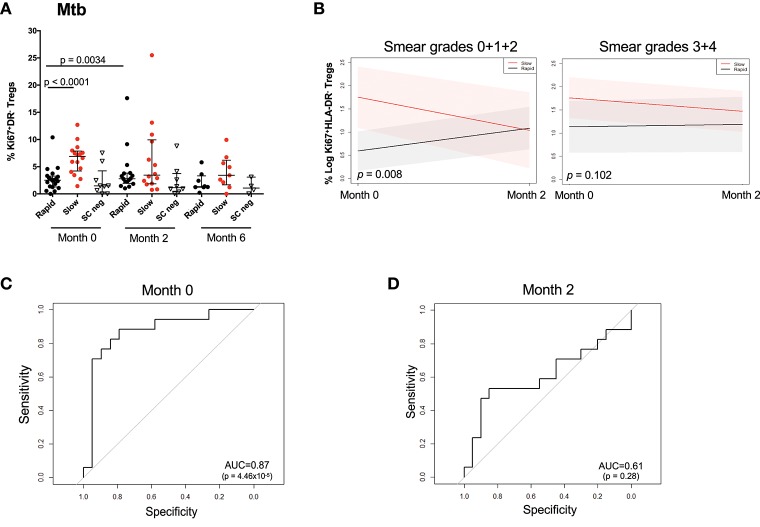
Changes in Mtb stimulated Ki67^+^HLA-DR^−^ Treg cells over the first 6 months of treatment and prediction of microbiological outcome. **(A)** Raw data points showing the difference between rapid (black), slow (red), and SC negative (inverted triangle) participants at months 0, 2, and 6. Differences between rapid and slow were assessed using the Mann–Whitney test. Solid lines represent the median and interquartile ranges in Mtb-stimulated patients. **(B)** Treg trajectory over the first 2 months of MDR-TB treatment for rapid and slow responders within low (0 + 1 + 2) and high (3 + 4) baseline smear grades groups. Black lines represent the modeled values for rapid responders and the red lines represent the modeled values for slow responders. The shaded area represents the 95% confidence intervals. *P*-values were derived from the mixed effect linear regression model using maximum likelihood estimation on the unimputed data, when baseline smear grades were factored into the model comprising microbiological outcome, visit and all two- and three-way interactions between the variables. **(C)** Receiver Operating Curve (ROC) analysis of Ki67^+^HLA-DR^−^ Treg cells after short-term Mtb stimulation at baseline (month 0). **(D)** Receiver Operating Curve (ROC) analysis of Ki67^+^HLA-DR^−^ Treg cells after short-term Mtb stimulation at 2 months of treatment.

Figure 4A shows the observed Treg frequencies over 6 months of treatment for the two responder groups and SC negative patients. At baseline, the rapid responding group possessed the same proportion of Ki67^+^HLA-DR^−^ Treg cells as the SC negative group, which remained unchanged throughout MDR-TB treatment. It is important to note that the proportion of Ki67^+^HLA-DR^−^ Treg cells declined with treatment, showing that the frequency of cells tracked with drug-induced clearance of bacilli. When the changes in Ki67^+^HLA-DR^−^ Tregs was modeled, the outputs approximated the observed profiles when BMI, age, and gender were included as covariates (Supplementary Figure 11). The confidence intervals did not overlap at baseline, indicating a significant difference between the rapid and slow responders at baseline (*p* = 0.0012) but this was no longer the case at the month 2 visit (*p* = 0.1599). Additionally, the temporal pattern of cell population changes differed between the two responding groups. While there is an increase in Treg frequency among the Rapid responders from baseline to month 2 and a decrease in Treg frequency among the Slow responders from baseline to month 2, neither of these changes are significant, (*p* = 0.1359, *p* = 0.2821, respectively). Similar results including the 6 months visit are included in Supplementary Figure 11. However, due to a larger number of missing values at month 6, these results should be interpreted with caution. Overall, slow responders showed a 33% decrease in Ki67^+^HLA-DR^−^ Treg cells and rapid responders showed a 12% decline. When we factored baseline smear grades into the model comprising microbiological outcome, visit and all two- and three-way interactions between the variables (Figure 4B), the group of patients with lower smear grade (0 + 1+ 2) showed a significant difference of Treg values between rapid and slow responders at baseline (*p* = 0.008) vs. the group of patients with higher baseline smear grade (3 + 4), where no differences (*p* = 0.102) were observed between responder groups. These findings corroborate the results reported in Figure 1F, where smear grade was significantly associated with the frequency of Treg cells and could be regarded as an effect modifier for the association of these cells between Rapid/Slow responders.

### Baseline Mtb and PPD-stimulated Ki67^+^HLA-DR^−^ treg cells can predict TCC

As there was a high frequency of Ki67^+^HLA-DR^−^ Treg cells at baseline in slow responders, we sought to identify whether baseline Mtb-stimulated Ki67^+^HLA-DR^−^ regulatory T cells could be useful predictors of TTC. ROC analysis of baseline frequencies of Mtb-stimulated Ki67^+^HLA-DR^−^ Treg cells showed a significant area under the curve (AUC) of 87% (*p* < 0.0001) with 81.2% sensitivity and 85% specificity (Figure 4C). This predictive power was not observed at 2 months after treatment initiation (Figure 4D).

## Discussion

This study has shown that a highly defined blood circulating T regulatory cell subset can distinguish, prior to second-line treatment for MDR-TB, the severity of disease and the pace at which patients respond to treatment. We used a whole blood method to measure T-cell mediated responses after a short-term re-stimulation with a cocktail of mycobacterium tuberculosis (TB7.7) antigen, early secreted antigenic target 6 (ESAT6) protein and culture filtrate protein 10 (CFP10) antigens. The strength of our study lies in the ability to link functional phenotypes of cells with meticulously measured clinical and microbiological outcomes. The frequencies of Mtb-stimulated CD3^+^CD4^+^CD25^hi^FoxP3^+^CD127^−^CD69^−^ Ki67^+^HLA-DR^−^ Treg were significantly elevated in patients who were SC positive vs. SC negative and in those patients with lung cavitation. The frequency of these cells were higher in the blood circulation in patients responding slowly to second-line MDR-TB treatment relative to those responding rapidly and could distinguish between fast and slow responders when measured prior to initiating therapy. Slow responders had higher Mtb-stimulated Ki67^+^HLA-DR^−^ Tregs compared to rapid responders when tracked longitudinally throughout treatment and the two groups showed different temporal trajectories. We speculate that the enhanced Ki67^+^HLA-DR^−^ Tregs in response to *in vitro* re-stimulation with Mtb antigens is a reflection of higher mycobacteria antigenic burden in slow responders.

Previous studies reported the distinction between HLA-DR^+^ and HLA-DR^−^ regulatory T cells (25). These authors demonstrated that MHC-II expression by human CD4^+^CD25^hi^ T cells identified a functionally distinct population of mature Treg and within the CD4^+^CD25^hi^ subset. HLA-DR expressing Treg cells can inhibit T cell proliferation and cytokine production via an early contact-dependent mechanism that is associated with induction of FoxP3. In striking contrast, HLA-DR^−^ CD4^+^CD25^hi^ T cells did not induce early contact-dependent suppression but were shown to initially enhance secretion of IL-10 and IL-4, and subsequently induce a late suppression of proliferation that was accompanied by Treg induction of FoxP3 expression. Furthermore, while both the HLA-DR^+^ Treg and HLA-DR^−^ Treg subsets can suppress via a cell contact-mediated mechanism, the HLA-DR^−^ Treg also can also suppress via secretion of IL-10. The intent of our study was not to seek the role of Treg cells, or mechanisms by which they function, but rather how these cells are related to disease and inflammatory status in MDR-TB. It is possible that Mtb-stimulated Ki67^+^HLA-DR^−^ Tregs might be a separate lineage within the CD4^+^CD25^hi^ subset, but we posit that these cells rapidly proliferate in response to higher levels of bacterial burden found in the MDR-TB patients responding slowly to antibiotic treatment.

The role of Treg cell populations in MTB disease is double-sided, as there is evidence for their role in the control of TB disease by dampening immune-mediated pathology, but at the same time Treg cells function by suppressing protective TB-stimulated immune responses and exacerbating active TB disease (26). For example, in diseased patients Treg cells have been shown to suppress T cell responses to mycobacterial antigens, whereas the CD4^+^CD25^hi^FoxP3^+^ cells of non-diseased individuals did not suppress the IFNγ secretion induced by protective mycobacterial antigens (12, 27). Our data suggest that this may also be the case as we found an inverse correlation between Ki67+HLA-DR^−^ Tregs and IFN-g-expressing activated CD4 and CD8 T cells suggesting that these cells have the ability to suppress Th1 responses in slow responders. *In vitro* co-culture experiments would provide this mechanistic link. There is also evidence that circulating mycobacterial-specific Treg cells can distinguish LTBI from active drug susceptible TB disease (28) and so serve as useful biomarkers. Serrano et al. (29) have also shown that CD4^+^CD25^hi^CD39^+^Foxp3^+^ Treg cells could successfully be used as a marker to distinguish between individuals being TST and quantiferon positive from those who were negative for both tests, thus showing the sensitivity of blood circulating Treg cells at discriminating the spectrum of LTBI.

Multiple studies have defined Treg cells using different markers, but most have shown a level of uniformity in being elevated in drug-susceptible and MDR-TB disease. In our study, we chose to extend a minimum panel set for Treg cells based on cancer immunotherapy prognosis (19). Our finding that elevated baseline proportions of Mtb-stimulated Ki67^+^HLA-DR^−^ Tregs in slow progressors and in patients with cavitation are in agreement with studies showing increased frequencies of FoxP3^+^CD25^+^ Tregs in MDR-TB patients (13) and in those with lung cavitation (30). Our data thus confirm that our defined population of Mtb-stimulated Ki67^+^HLA-DR^−^ Treg cells can be used as a potential marker of disease severity and is proportionate to the state of host inflammation.

It was evident from our data that high bacillary load (as measured by grades of smear scores and prolonged TCC), was associated with cavitation and a greater frequency of circulating Mtb-stimulated Ki67^+^HLA-DR^−^ Treg cells in slow responders. Although CD4^+^CD25^hi^FoxP3^+^Treg cells are naturally generated in the thymus (31, 32) they can also be induced in the periphery, in both mice (33) and humans (34), suggesting that peripheral Treg cells may arise from antigenic challenge during the course of an immune response (35). Treg cells have been shown, after MTB infection, to be derived from a population of pre-existing Tregs in the draining lymph nodes of the lungs (36) and that mycobacterial antigens have been shown to induce the expansion of CD4^+^CD25^hi^FoxP3^+^ Treg cells (37, 38). Our results show that the majority of the detectable Mtb-stimulated Ki67^+^HLA-DR^−^ Treg are in the early differentiated memory stage of maturation and that they possibly function to diminish protective CD4+ and CD8+ T cell immunity and are possibly expanding by ensuing high bacillary load in the lung. We have previously shown that lung radiological severity was accompanied by high levels of inflammatory markers in the plasma from some of the same patients in the cohort (18), suggesting that high bacillary load results in inflammatory signals. Our finding that there were no differences in Ki67^+^HLA-DR^−^ Tregs between rapid responders and SC—patients at baseline and throughout 6 months of treatment also supports the speculation that these cells arise in the circulation in response to high levels of antigenic stimulation—as when actively dividing bacilli are cleared or very low, in the case of SC—, Treg cells constitute a small proportion of circulating cells. Collectively, our data support the double-sided nature, and counter-intuitive role, of Treg in TB disease: that these cells likely suppress specific protective immunity to TB and allow unchecked bacterial replication and enhanced inflammation leading to lung pathology.

These potential mechanistic links between Treg cells and disease severity in MTB is supported by experimental studies in mice showing that temporary depletion of Tregs resulted in lower bacterial burden in the lungs followed by reduced pathological conditions (39).

Whatever the mechanisms of Treg cells in our MDR-TB patients, we portend that the increased ability of single IFNg-expressing activated CD4 and CD8 T cells to exclusively express IFNg in rapid responders vs. slow responders may mirror the lower bacillary load and CRP/SAA status in these patients. The inverse relationship between IFNg-expressing activated Mtb-stimulated CD4 and CD8 T cells with Ki67^+^HLA-DR^−^ Tregs may be explained by the suppressive functional association between these cells and T cell activation, where levels of Th1-type mediators may be rendered dysfunctional by high levels of Ki67^+^HLA-DR^−^ Treg cells. Previous data has shown strong associations between TNFa concentrations in BAL fluid and lung involvement (40), and our longitudinal results show that after 6 months of second-line therapy, TNFα expression by Mtb-activated CD4 T cells persisted in slow responders (data not shown). Thus, our observed slower rate of Ki67^+^HLA-DR^−^ Treg cell frequency decline in MDR-TB patients who were relatively unresponsive to treatment in the first few weeks, confirms the relationship between the proportion of these cells with bacillary load and likely continuous inflammation. Our data suggest that it is likely that inflammation may be driven by bacillary load, as when high smear grade scores were included in the model, no differences in Treg frequencies at baseline were observed. In those patients rapidly responding to treatment, there was a temporal, but transient, rise in Treg cells at 8 weeks after initiating treatment, in agreement with observations made previously (41). Whatever the cause of the increased frequencies of Treg cells in rapid responders in the first 2 months, the resulting ability of these cells to predict TCC after initiation of second-line drugs fell away.

In conclusion, baseline frequencies of Mtb-stimulated Ki67^+^HLA-DR^−^ Tregs in blood represent a good predictor of microbiological outcome in MDR-TB patients. The ROC curve analysis revealed a powerful ability of this marker to predict at baseline microbiological outcome with 81% sensitivity and 85% specificity and the loss of this predictive power after 2 months of treatment was due to the increased frequency of Ki67^+^HLA-DR^−^ Tregs in the rapid responders. It is likely that the inflammatory milieu in the slow responding patients drives the expansion of suppressive Treg, which in turn may lower the ability of host immunity to effect bacillary clearance. Whatever the cause and effect, the frequency of blood circulating Mtb-stimulated CD4^+^ Ki67^+^HLA-DR^−^ Treg cells prior to MDR-TB second line treatment can be used to predict microbiological outcome.

## Author contributions

GK, DF, FC, and CG designed the study. SF, GK, DF, and CG wrote the manuscript. SF ran the experiments, performed data analysis and statistical testing. SV and NI oversaw the quality and interpreted the microbiological outcome data. MR and FL performed the statistical analysis, interpretation of the data, and wrote parts of the manuscript.

### Conflict of interest statement

The authors declare that the research was conducted in the absence of any commercial or financial relationships that could be construed as a potential conflict of interest.
