# Supplementary material for: Frequency of Circulating CD4+Ki67+HLA-DR− T Regulatory Cells Prior to Treatment for Multidrug Resistant Tuberculosis Can Differentiate the Severity of Disease and Predict Time to Culture Conversion
Source: Front Immunol. 2018 Oct 25;9:2438. doi: 10.3389/fimmu.2018.02438 (PMC6209685; doi:10.3389/fimmu.2018.02438)
Supplement: Supplementary file 1 [file Data_Sheet_1.pdf]

Supplementary Material

Frequency of circulating $CD4^{+}Ki67^{+}HLA\text{-}DR^{-}$ T regulatory cells prior to treatment for Multidrug Resistant tuberculosis can differentiate the severity of disease and predict Time to Culture Conversion.

Selena Ferrian[1+], Melinda Ross[2], Francesca Conradie[3], Shaheed Vally Omar[4], Nazir Ismail[4,5], Francesca Little[2], Gilla Kaplan[6], Dorothy Fallows[6], Clive M Gray[1,7*].

1. Institute of Infectious Disease and Molecular Medicine, Division of Immunology, Department of Pathology, University of Cape Town, South Africa
2. Department of Statistical Sciences, University of Cape Town, South Africa
3. Faculty of Health Sciences, University of the Witwatersrand, Johannesburg, South Africa
4. Centre for Tuberculosis, National Institute of Communicable Diseases, Sandringham, Johannesburg, South Africa 2131
5. University of Pretoria, Department of Medical Microbiology, Pretoria, South Africa
6. Public Health Research Institute, Rutgers University, Newark, NJ, United States
7. National Health Laboratory Services, Cape Town, South Africa

\+ Current address: Department of Pathology, Stanford University, Palo Alto, California, USA

- Correspondence: Clive M Gray, Institute of Infectious Disease and Molecular Medicine, Division of Immunology, Department of Pathology, University of Cape Town, South Africa; clive.gray@uct.ac.za

Keywords: Treg cells, Mycobacteria tuberculosis, Multi-drug resistant tuberculosis, lung cavities, sputum culture, smear grades, culture conversion.

Supplementary Figure 1: (A, B) Representation of the flow cytometry gating strategy showing area versus height forward scatter (FSC) to gate singlets and then gate around lymphocytes. The time gate was used to identify smooth flow of cells and red polygons and boxes represent gates used to select populations of cells to be characterized further. TD: terminal differentiation; LD: late differentiation; ED: early differentiation. (C) Representation of the gating strategy used to identify IL-2 and IFNg expression in CD69+ Treg. Light and boldened blue boxes represent CD69 negative and positive, respectively.

Supplementary Figure 2: (A) List of antibodies, fluorescent dyes, clones, isotypes and vendors used to make up the polychromatic panel. Areas shaded according to whether antibodies were used for surface or intracellular staining. (B) Fluorescence minus one (FMO) for Ki67 in $CD3^{+}CD4^{+}$ T cells.

Supplementary Figure 3: A mosaic plot showing the comparison between baseline cavitation and sputum smear Acid Fast Bacilli (AFB) grades, with green depicting absence of cavitation and purple depicting presence of cavitation, within slow and rapid responders to MDR-TB treatment (n=42). The Fisher's exact test was used for statistical correlation

between microbiological outcome and smear grades or cavitation. Cavitary lesions were present in 28 (74%) of the patients with available chest X-ray reviews.

0= Absence of AFB; 1= scanty; 2= AFB+; 3=AFB++; 4+AFB+++

NA= Not available

Supplementary Figure 4:

Correlation between $Ki67^{+}HLA\text{-}DR^{-}$ Treg cells and Log_{10} time to culture conversion (TCC) without stimulus in culture (NS) and after PHA stimulation. Black symbols represent rapid responders and red symbols represent slow responders. MITO = mitogen (PHA).

Supplementary Figure 5: Comparison of Treg cells between SC negative (light shaded circles) and SC positive (dark solid circles) patients under each stimulation condition. NS = no stimulus; Mtb = mycobacterium tuberculosis; PPD = purified protein derivative; MITO = mitogen (PHA). The horizontal line represents the median values ± interquartile ranges (IQR). Statistical comparisons between responding patients were made using the Mann-Whitney U test. Statistical comparisons within the same group of patients over time were made using Wilcoxon matched-pairs test.

Supplementary Figure 6: Comparison of Mtb-stimulated $Ki67^{-}HLA\text{-}DR^{-}$ double negative Treg cell frequencies between SC negative (light shaded circles) and SC positive (dark solid circles). The horizontal line represents the median values ± interquartile ranges (IQR). Statistical comparisons between responding patients were made using the Mann-Whitney U test. Mtb = mycobacterium tuberculosis.

Supplementary Figure 7: Comparison of Mtb-stimulated Ki67-HLA-DR- double negative Treg cell frequencies between slow responders (<71 days) and rapid responders (>71 days). The horizontal line represents the median values ± interquartile ranges (IQR). Statistical comparisons between responding patients were made using the Mann-Whitney U test. Mtb = Mycobacterium tuberculosis.

Supplementary Figure 8: Early Differentiated (ED) memory CD4+ $Ki67^{+}$HLA-DR- Treg cells and the relationship between microbiological outcome. A. Comparison of ED ($CD27^{+}CD45RA^{-}$) Mtb-stimulated $Ki67^{+}HLA\text{-}DR^{-}$ Treg cells between fast (<71 days, n=20) and slow (>71 days, n=16) responders; black solid symbols represent fast responders and red symbols represent slow responders. Six patients were excluded due to either not enough $Ki67^{+}HLA\text{-}DR^{-}$ Treg cells in the analysis gate or missing samples (n=1). B. Comparison of ED ($CD27^{+}CD45RA^{-}$) PPD-stimulated $Ki67^{+}HLA\text{-}DR^{-}$ Treg cells between fast (<71 days, n=13) and slow (>71 days, n=9) responders; black solid symbols represent fast responders and red symbols represent slow responders. Twenty patients were excluded due to either not enough $Ki67^{+}HLA\text{-}DR^{-}$ Treg cells in the analysis gate or missing samples (n=10). C & D: Pearson correlation between ED ($CD27^{+}CD45RA^{-}$) Mtb- and PPD- stimulated $Ki67^{+}HLA\text{-}DR^{-}$ Treg cells with $\log_{10}$ TCC.

Supplementary Figure 9: Pearson correlation between % Log_{10} bulk Treg cells and Log_{10} time to culture conversion (TCC) in response to Mtb and PPD stimulus. Black symbols represent rapid responders and red symbols represent slow responders. Mtb = mycobacterium tuberculosis; PPD = purified protein derivative.

Supplementary Figure 10: Model diagram summarizing the correlation between response to MDR-TB treatment and the frequency of total Tregs and $Ki67^{+}HLA\text{-}DR^{-}$ Tregs. The red boxed area denotes slow response to treatment and the black boxed area denotes fast response to treatment. Numbers indicate the median values of $Ki67^{+}HLA\text{-}DR^{-}$ Treg cells ± interquartile ranges (IQR) within each responder group. The bottom legend indicates the median time (71 days) to culture conversion, which allow the stratification of patients as fast and slow responders to MDR-TB treatment.

Supplementary Figure 11: Longitudinal analysis of $\log_{10}$ transformed Mtb-stimulated $Ki67^{+}HLA\text{-}DR^{-}$ Treg frequencies from baseline (month 0), month 2 and month 6 of MDR-TB treatment. Black solid and dotted lines represent the mean observed and modeled values, respectively, for rapid responders. Red solid and dotted lines represent the mean observed and modelled values, respectively, for slow responders. The shaded area represents the 95% confidence intervals. P-values were derived from the mixed effect linear regression model using maximum likelihood estimation on the unimputed data, with adjustment for the following covariates: age, gender, and BMI.

A

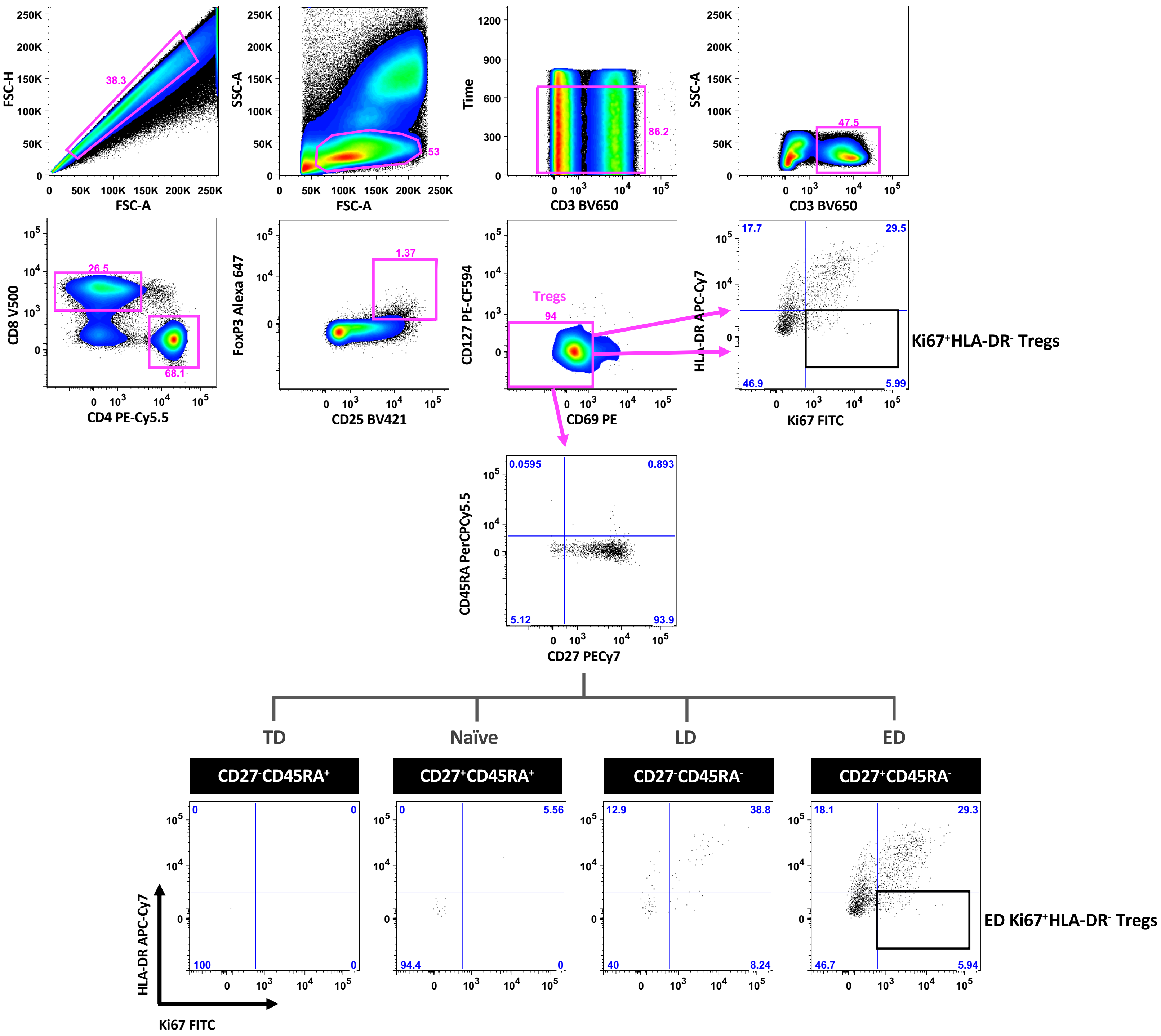

FSC-H
FSC-A
38.3
SSC-A
FSC-A
53
Time
CD3 BV650
86.2
SSC-A
CD3 BV650
47.5
CD8 V500
CD4 PE-Cy5.5
26.5
68.1
FoxP3 Alexa 647
CD25 BV421
1.37
CD127 PE-CF594
CD69 PE
Tregs
94
HLA-DR APC-Cy7
Ki67 FITC
17.7
29.5
46.9
5.99
Ki67+HLA-DR- Tregs
CD45RA PerCPCy5.5
CD27 PECy7
0.0595
0.893
5.12
93.9
TD
CD27-CD45RA+
Naïve
CD27+CD45RA+
LD
CD27-CD45RA-
ED
CD27+CD45RA-
HLA-DR APC-Cy7
Ki67 FITC
ED Ki67+HLA-DR- Tregs

SF1

B

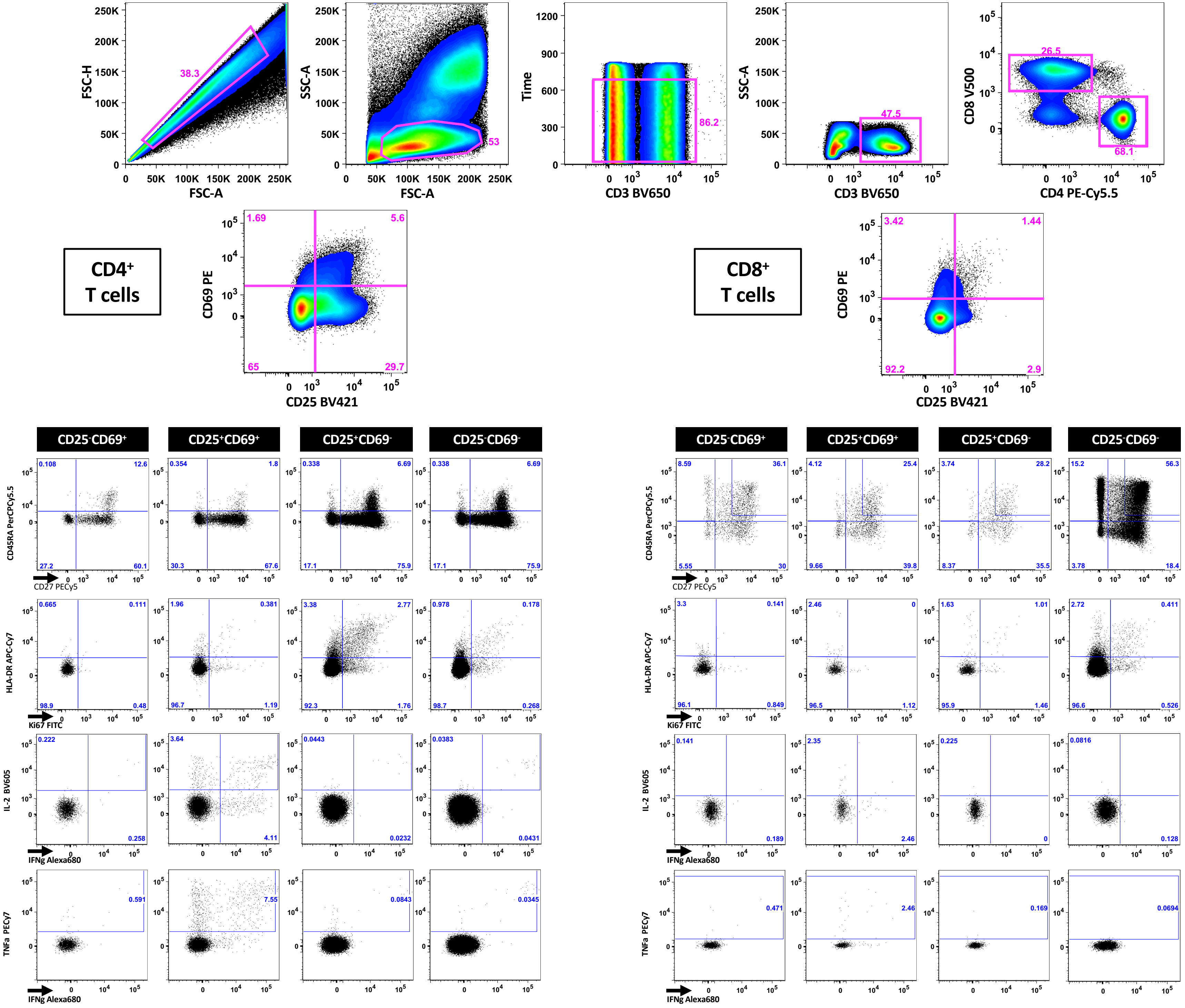
FSC-H
FSC-A
38.3
SSC-A
53
Time
CD3 BV650
86.2
47.5
CD8 V500
CD4 PE-Cy5.5
26.5
68.1
CD4+ T cells
CD69 PE
CD25 BV421
1.69
5.6
65
29.7
CD8+ T cells
3.42
1.44
92.2
2.9
CD25-CD69+
CD25+CD69+
CD25+CD69-
CD25-CD69-
CD45RA PerCPCy5.5
CD27 PECy5
HLA-DR APC-Cy7
Ki67 FITC
IL-2 BV605
IFNg Alexa680
TNFa PECy7

SF1

C

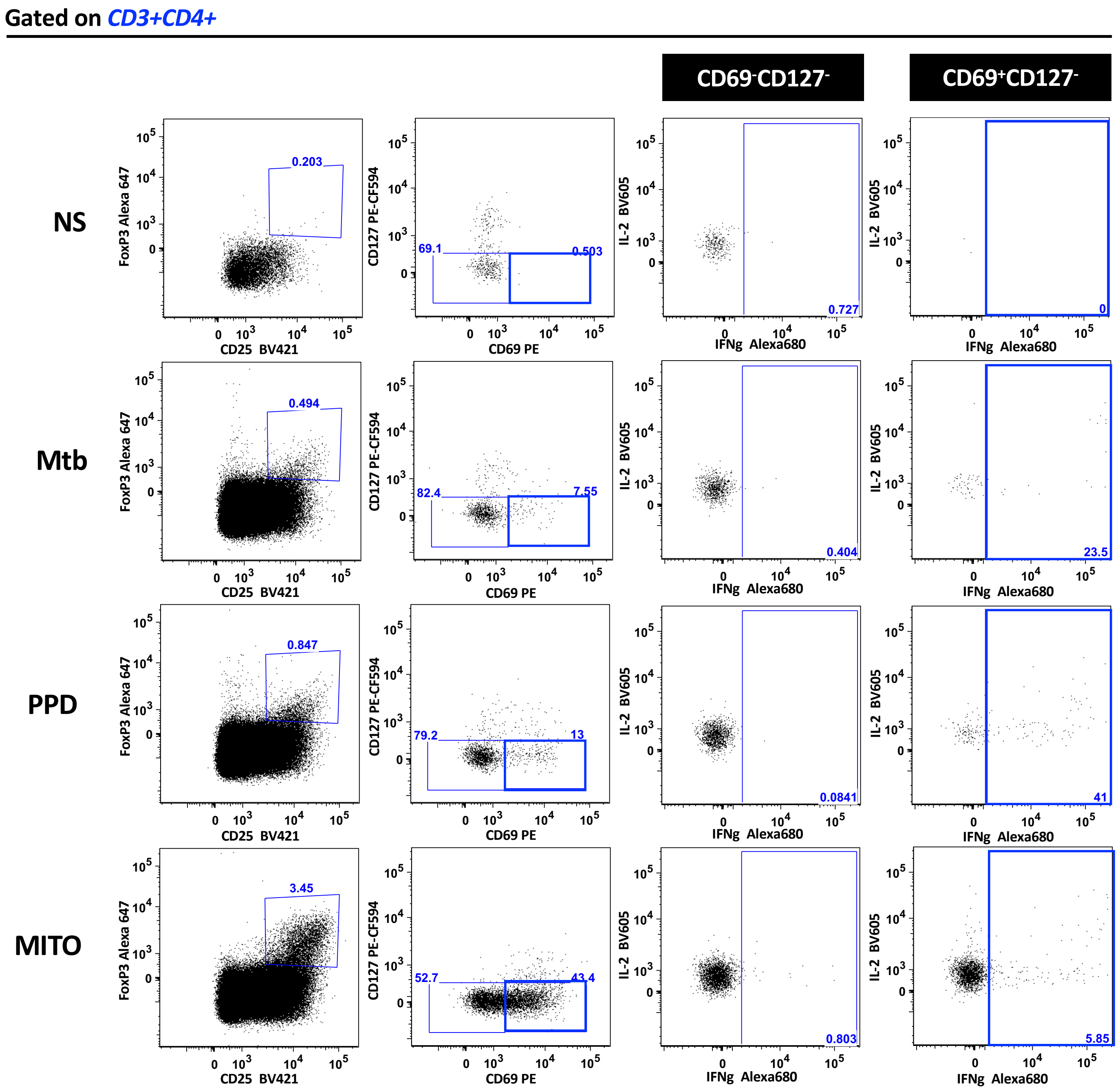
Gated on CD3+CD4+
CD69-CD127-
CD69+CD127-
NS
Mtb
PPD
MITO
FoxP3 Alexa 647
CD25 BV421
CD127 PE-CF594
CD69 PE
IL-2 BV605
IFNg Alexa680
0.203
69.1
0.503
0.727
0
0.494
82.4
7.55
0.404
23.5
0.847
79.2
13
0.0841
41
3.45
52.7
43.4
0.803
5.85

SF1

SF2

A

Ref.#	Antibody	Fluorescence	Clone	Isotype	Company
317324	Anti-human CD3	BV650	OKT3	Mouse IgG2a,k	Biolegend
MHCD0418	Anti-human CD4	PECy5.5	S3.5	Mouse IgG2a	Invitrogen
560774	Anti-human CD8	V500	RPA-T8	Mouse IgG1,k	BD Biosciences
562442	Anti-human CD25	BV421	M-A251	Mouse IgG1,k	BD Biosciences
15-0279	Anti-human CD27	PECy5	O323	Mouse IgG1,k	eBiosciences
563429	Anti-human CD45RA	PerCP-Cy5.5	HI100	Mouse IgG2b,k	BD Biosciences
341652	Anti-human CD69	PEGreen	L78	Mouse IgG1,k	BD Biosciences
562397	Anti-human CD127	PE-CF594	HIL-7R-M21	Mouse IgG1,k	BD Biosciences
307618	Anti-human HLA DR	APC-Cy7	L243	Mouse IgG2a,k	Biolegend
557995	Anti-human IFNγ	Alexa700	B27	Mouse IgG1,k	BD Biosciences
557647	Anti-human TNFα	PECy7	MAb11	Mouse IgG1,k	BD Biosciences
556026	Anti-human Ki67	FITC	B56	Mouse IgG1,k	BD Biosciences
563947	Anti-human IL-2	BV605	5344.111	Mouse IgG1,k	BD Biosciences
560045	Anti-human FoxP3	Alexa647	259D/C7	Mouse IgG1	BD Biosciences
562574	Transcription Factor Buffer Set				BD Biosciences
552843	Compensation Bead Anti-mouse Ig,k				BD Biosciences
563794	Brilliant Stain buffer				BD Biosciences

Surface staining   Intracellular staining   Other reagents

B

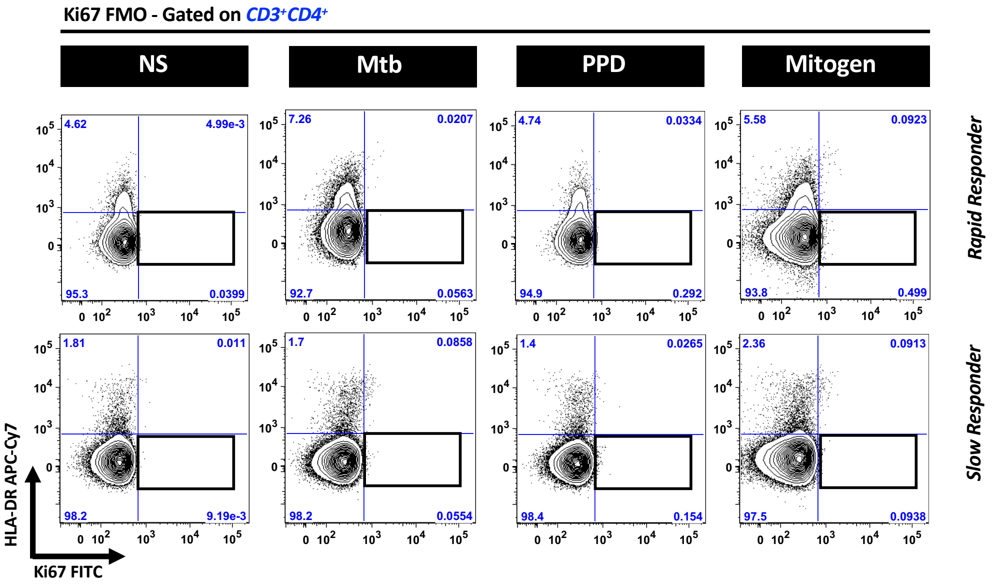

SF3

Inside each box is the number of patients.

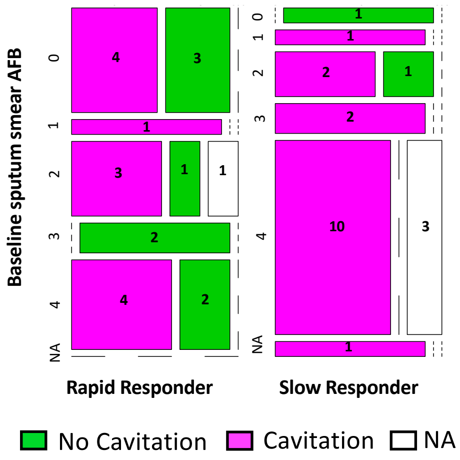

SC-positive patients (N=42)	**Microbiological Outcome**
**Baseline Cavitation**	p = 0.0673 Fisher's exact test
**Baseline Sputum Smear AFB**	p = 0.0071; r = 0.4142

SF4

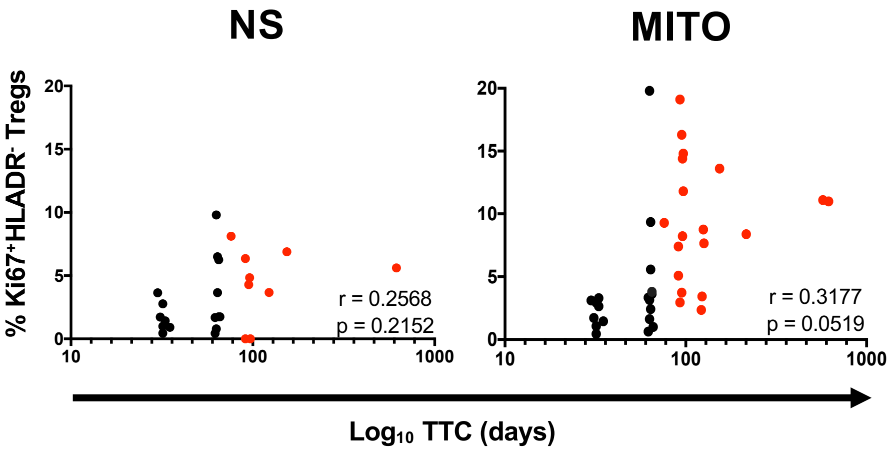
NS
MITO
% Ki67+HLADR- Tregs
20
15
10
5
0
10
100
1000
r = 0.2568
p = 0.2152
r = 0.3177
p = 0.0519
Log10 TTC (days)

SF5

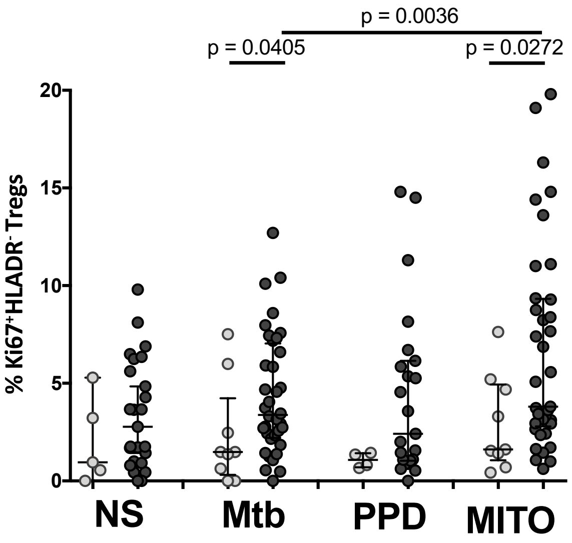
p = 0.0036
p = 0.0405
p = 0.0272
20
15
10
5
0
% Ki67+HLADR- Tregs
NS
Mtb
PPD
MITO

SF6

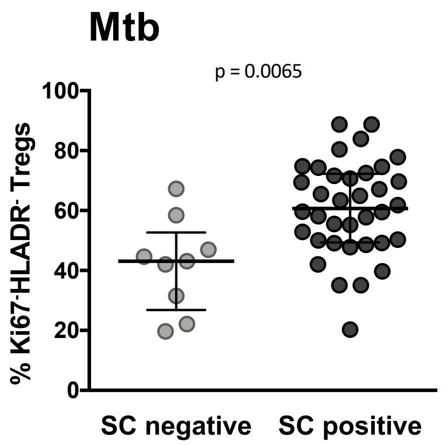

Mtb
p = 0.0065
% Ki67-HLADR- Tregs
100
80
60
40
20
0
SC negative
SC positive

SF7

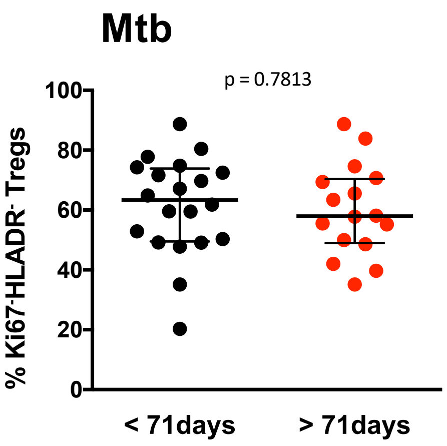

SF8

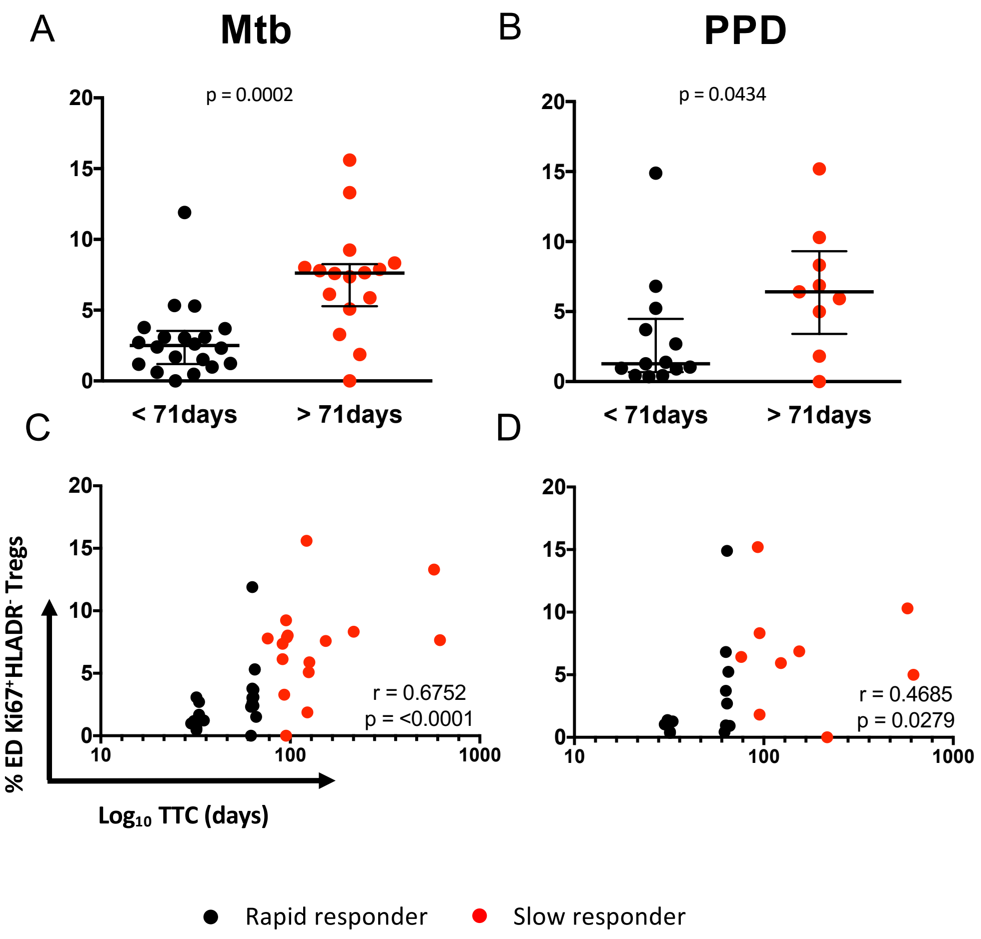

A
Mtb
p = 0.0002
20
15
10
5
0
< 71days
> 71days
B
PPD
p = 0.0434
20
15
10
5
0
< 71days
> 71days
C
% ED Ki67+HLADR- Tregs
20
15
10
5
0
10
100
1000
Log10 TTC (days)
r = 0.6752
p = <0.0001
D
20
15
10
5
0
10
100
1000
r = 0.4685
p = 0.0279
Rapid responder
Slow responder

SF9

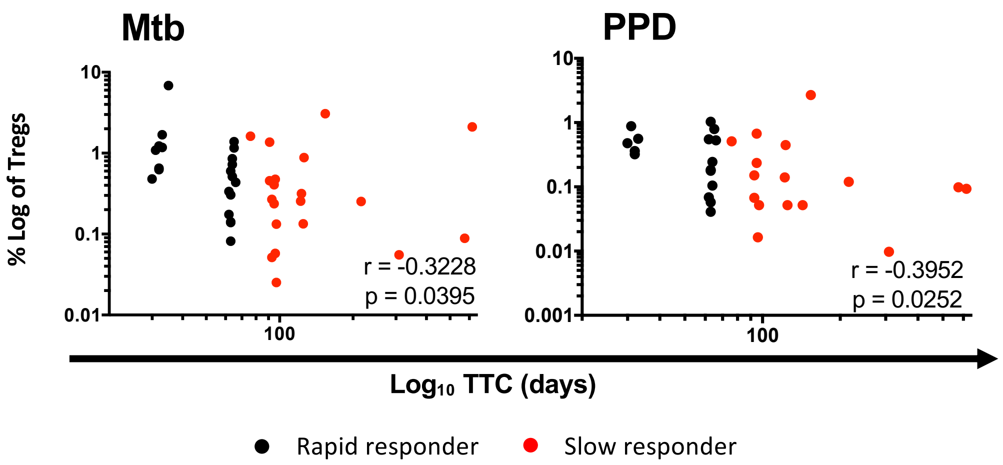
Mtb
PPD
% Log of Tregs
10
1
0.1
0.01
0.001
100
r = -0.3228
p = 0.0395
r = -0.3952
p = 0.0252
Log10 TTC (days)
Rapid responder
Slow responder

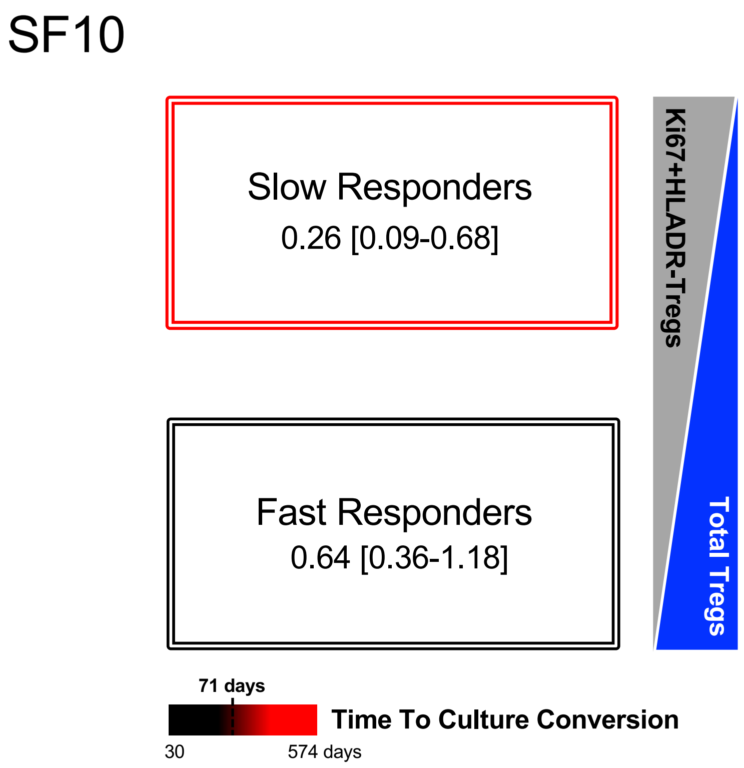
SF10
Slow Responders
0.26 [0.09-0.68]
Fast Responders
0.64 [0.36-1.18]
Ki67+HLADR-Tregs
Total Tregs
71 days
30
574 days
Time To Culture Conversion

SF11

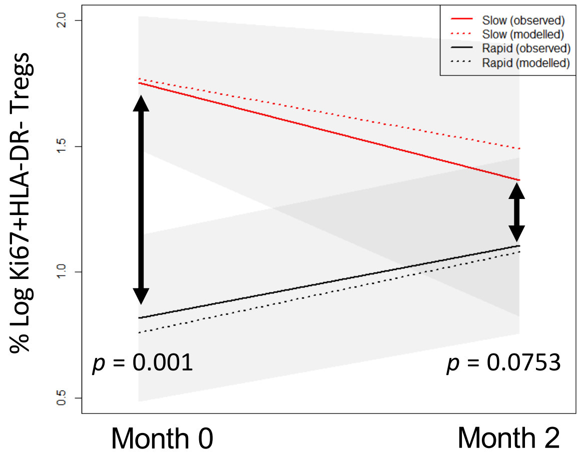

% Log Ki67+HLA-DR- Tregs
Slow (observed)
Slow (modelled)
Rapid (observed)
Rapid (modelled)
p = 0.001
p = 0.0753
Month 0
Month 2

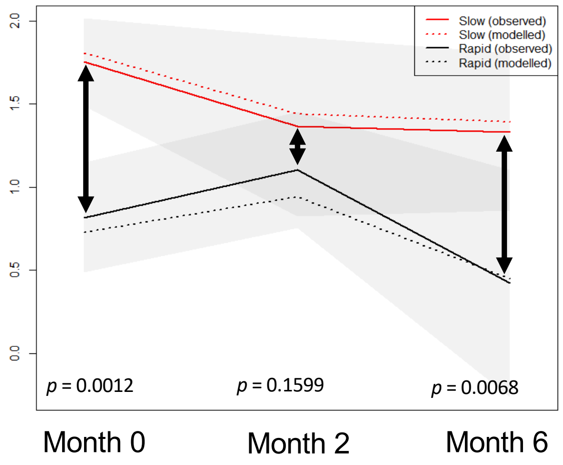

Slow (observed)
Slow (modelled)
Rapid (observed)
Rapid (modelled)
p = 0.0012
p = 0.1599
p = 0.0068
Month 0
Month 2
Month 6
